# Ecological networking of cystic fibrosis lung infections

**DOI:** 10.1038/s41522-016-0002-1

**Published:** 2016-12-02

**Authors:** Robert A Quinn, Katrine Whiteson, Yan Wei Lim, Jiangchao Zhao, Douglas Conrad, John J LiPuma, Forest Rohwer, Stefanie Widder

**Affiliations:** 1 Department of Biology, San Diego State University, San Diego, CA 92182 USA; 2 Department of Molecular Biology and Biochemistry, University of California Irvine, Irvine, CA 92697 USA; 3 Department of Pediatrics and Communicable Diseases, University of Michigan Medical School, Ann Arbor, MI 48109 USA; 4 Department of Medicine, University of California at San Diego, La Jolla, CA 92037 USA; 5 CUBE, Department of Microbiology and Ecosystem Science, University of Vienna, Althanstr.14 A-1090, Vienna, Austria; 6 Skaggs School of Pharmacy and Pharmaceutical Sciences, University of California at San Diego, La Jolla, CA 92093 USA; 7 Division of Agriculture, Department of Animal Science, University of Arkansas, Fayetteville, AR 72701 USA; 8 CeMM - Research Center, for Molecular Medicine of the Austrian Academy of Sciences, Lazarettg, 14, A-1090 Vienna, Austria

## Abstract

In the context of a polymicrobial infection, treating a specific pathogen poses challenges because of unknown consequences on other members of the community. The presence of ecological interactions between microbes can change their physiology and response to treatment. For example, in the cystic fibrosis lung polymicrobial infection, antimicrobial susceptibility testing on clinical isolates is often not predictive of antibiotic efficacy. Novel approaches are needed to identify the interrelationships within the microbial community to better predict treatment outcomes. Here we used an ecological networking approach on the cystic fibrosis lung microbiome characterized using 16S rRNA gene sequencing and metagenomics. This analysis showed that the community is separated into three interaction groups: Gram-positive anaerobes, *Pseudomonas aeruginosa,* and *Staphylococcus aureus*. The *P. aeruginosa* and *S. aureus* groups both anti-correlate with the anaerobic group, indicating a functional antagonism. When patients are clinically stable, these major groupings were also stable, however, during exacerbation, these communities fragment. Co-occurrence networking of functional modules annotated from metagenomics data supports that the underlying taxonomic structure is driven by differences in the core metabolism of the groups. Topological analysis of the functional network identified the non-mevalonate pathway of isoprenoid biosynthesis as a keystone for the microbial community, which can be targeted with the antibiotic fosmidomycin. This study uses ecological theory to identify novel treatment approaches against a polymicrobial disease with more predictable outcomes.

## Cystic fibrosis: the role of microbial ecology

Studying the varying ecology of complex microbial infections in the lungs may help guide treatment options in cystic fibrosis. The disease is complicated due to infection with a diverse community of pathogenic microorganisms. The success of treatments based on identifying and targeting specific microbes can be unpredictable due to the effects of interactions within the mixed microbial ecosystem. Stefanie Widder at the University of Vienna together with Robert Quinn and co-workers in the USA, studied these interactions in six specific cases of cystic fibrosis using lung infection samples gathered over 10 years. They identified three main groups of bacteria involved and developed several hypotheses about inter-relationships that can affect the success of antibiotic treatments. The researchers argue that their new hypotheses and overall ‘ecological theory’ will lead to more-reliable predictions of the likely benefits of specific treatment regimes.

## Introduction

Many infections are polymicrobial in nature. Complex assemblages of bacteria, fungi, and viruses are the norm in cutaneous infections and many chronic diseases of the digestive system, the oral cavity, and the airways. This is exemplified in the inherited multi-system disorder cystic fibrosis (CF), where a complex community of microbes colonizes the lungs, adapts to the lung environment, and develops an ecological community of organisms interacting with each other and the host.^1–3^ Treatment approaches to polymicrobial infections do not differ much from single pathogen infections, where isolates of the infected area are obtained and then screened for antibiotic resistance to determine which drugs are chosen for therapy. This approach discounts that the isolated strains exist in an ecological community, characterized by interdependencies and competition between its constituents. Not surprisingly, the physiology and antimicrobial susceptibility of isolates in vitro does not always translate to the complex infection in vivo, where the drugs are meant to act.^4^ Synergisms, antagonisms, and complex community dynamics all occur in these systems, complicating treatment efforts against particular pathogens.^5^ New approaches with a greater likelihood of treatment success are required that target a microbiome collectively, taking into account its physiology and community structure.

A community ecology approach to CF has been proposed since the understanding that the lungs contain a more diverse microbial community than that revealed by routine culturing methods.^6–9^ Lung microbiome studies have provided detailed insight into the microbial diversity of CF lung infections,^7, 10–13^ however, clinical practice has not changed significantly in light of this broadly accepted understanding. Models of microbiome dynamics are needed to aid in clinical translation of microbial ecology findings from the laboratory. The Climax and Attack Model (CAM) was first proposed to bring together our increasing knowledge about CF lung microbial diversity and community ecology.^1^ The model was originally focused on CF pulmonary exacerbations (CFPE), which are events of acute disease where patients present to clinicians with increased symptoms requiring more aggressive treatment.^14^ These events often result in hospitalization and greatly increase patient morbidity and health care costs.^15^ The CAM is founded on empirical evidence showing that similar bacterial communities exist during CFPE and times of stable disease;^16–19^ thus, it focuses on functional changes in community activity. It proposes that microbial functional behavior changes as patients develop an unstable community associated with CFPE, and is based on fundamental properties of classical ecology, including the existence of alternative stable states within an ecosystem.^20–23^ Recently, a study attempted to test the CAM by growing the CF microbial community from clinical samples in an environment mimicking the CF lung and monitoring microbial physiology.^24^ It demonstrated that functional changes in core community physiology do occur during the development of a CFPE, characterized by an increase in microbial fermentation. Further investigations into the drivers of CF microbiome dynamics and their relationship to the CAM will allow for better translation of an ecological understanding of CF microbiology into novel treatments for lung infections.

Bacterial ribosomal RNA gene sequencing (16S rRNA) has provided a detailed catalog of microbial taxa in CF lungs.^6, 7, 12^ Longitudinal studies through CFPE have shown mixed results, some studies describe significant changes in microbial profiles associated with disease state,^18, 19, 24–26^ and others do not.^27, 28^ Metagenomic sequencing has been less commonly employed, but is an even more powerful approach, as it allows for more detailed taxonomic information along with functional annotations.^29, 30^ Although these methods are useful for analyzing the structure and function of microbial communities, translating high-throughput sequencing data into predictive models with clinical relevance requires tools to identify patterns in the data relevant to disease. Aiming beyond purely compositional markers, microbial community structure can be predicted by network analysis.^31–33^ Using graph-topological arguments, such networks can be used to predict keystone species, members of the community that have a disproportionate influence on the overall community structure regardless of their abundance.^34^ Furthermore, networks enable the link between community structure and observed host phenotypes.^35, 36^ This approach to a pathogenic microbiome focuses on the ecological interdependencies within the microbial ecosystem, generates testable hypotheses on novel targets for treatment, and provides a global view of polymicrobial infection to identify its potential weak points. Application of network analysis to high-throughput sequencing data is a valuable means of analyzing complex data sets to better facilitate their translation to a clinical setting.

Here we use co-occurrence networking analysis of taxonomic and functional annotations of DNA sequence data from the CF lung microbiome to investigate its ecological structure as inferred across many patients. With attention to keystone species and pathways, the networking approach revealed that the structure of the microbial community reflects the Climax and Attack Model. In addition, metagenomic reads from keystone pathways were mapped back to their bacterium of origin to reveal the drivers of pathways crucial to overall community structure. This analysis revealed a novel drug target for CF and which bacteria it is likely to affect. We conclude that the CAM model is separated not only taxonomically, but also metabolically, based on amino acids or sugars as the principle carbon source. This study demonstrates the utility of networking approaches to identify the structure in microbial communities and how the detection of keystone features can reveal functional and taxonomic weak points as targets for polymicrobial infections.

## Results

### Structure of the CF lung microbiome

**Fig. 1:**
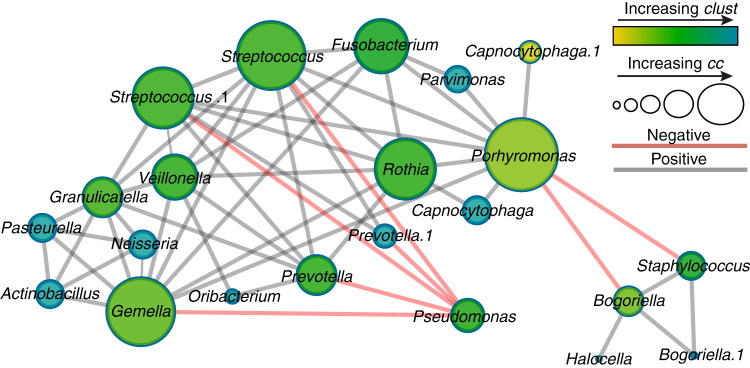
Co-occurrence network inferred from the SparCC algorithm applied to 16S rRNA gene data of 126 CF sputum samples. Co-occurrence and anti-occurrence of taxa is denoted with grey lines and red lines, respectively. Nodes are sized by their degree closeness centrality (cc) and colored by increasing clustering coefficient (increasing from olive to green to blue). The major taxa clustering strongly support the CAM hypothesis, stating that the CF community splits into two differently pathogenic subgroups: Attack Community members such as *Streptococcus*, *Veillonella* and *Porphyromonas*, and the Climax Community dominated by *Pseudomonas* and *Staphylococcus*

To gain insight into the microbial community structure in CF lungs, we created a co-occurrence network^37^ (referred to as ‘OTU (operational taxonomic unit) network’) from previously published 16S rRNA gene microbiome data from 6 patients collected over 10 years.^38^ This data set represents a decade long longitudinal study of how the microbial communities of a CF lung infection change. Our OTU network was used to visualize the interrelationships within the CF lung as disease progresses. The data set was queried for co-presence or absence patterns between particular microbes. For every statistically significant event (*p* < 0.01, correlation >|0.2|), an edge between them was accepted. The topology of the inferred network (nodes = 22, edges = 56) revealed three main clusters that are separated by anti-correlations, but displayed only positive correlations within the cluster (Fig. 1).

The first cluster is a complex group of 17 positively interacting operational taxonomic units (OTUs, binned at 97 % sequence identity) comprised of mostly Gram-positive anaerobes, including *Prevotella* and *Parvimonas,* and Gram-negative anaerobes, such as *Veillonella*, *Porphyromonas and Fusobacterium* (Fig. 1). Also contained in this group were the facultative anaerobes *Streptococcus, Granulicatella* and *Rothia*, the obligately fermentative *Gemella*, and a satellite subgroup containing members of the Pasteurellaceae and *Neisseria*. Except for this satellite, common among these group members is a core anaerobic physiology representing a functionally adapted cluster with similar metabolism. The other two significant clusters were much smaller, one containing *Pseudomonas* as a singleton, and the other, *Staphylococcus*, *Halocella* and *Bogoriella*. The latter two groups displayed anti-correlations with the first anaerobic group. *Pseudomonas* displayed negative correlations against specifically *Gemella, Streptococcus, Porphyromonas* and *Prevotella*, while the *Staphylococcus* group negatively interacts only with *Porphyromonas*. Predicted keystone bacterial OTUs were primarily anaerobes including *Streptococcus* spp., *Prevotella*, *Veillonella*, *Fusobacterium* and *Gemella* when both positive (Table 1) and negative interactions were included in the prediction (Supplementary Table S1).^37^

**Table 1:** Predicted keystoneness of OTUs in taxa co-occurrence NWs based on data by Zhao et al.^38^

OTU	k	Closeness centrality (cc)	Clustering coefficient (clust)	Keystone rank
*Streptococcus*.1	3.7	0.17	0.45	1
*Prevotella*	3.25	0.14	0.5	2
*Streptococcus*	3.75	0.14	0.36	3
*Veillonella*	2.8	0.13	0.86	3
*Fusobacterium*	2.7	0.13	0.67	4
*Gemella*	4	0.1	0.29	5
*Granulicatella*	2.75	0.1	0.5	6
*Bogoriella*.1	1.75	0.08	0.67	7
*Halocella*	1.3	0.07	1	8
*Prevotella*.2	1	0.14	–	9
*Bogoriella*	2.25	0.08	0.22	10
*Pseudomonas*	3	0.06	0.1	11
*Bordatella*	1	0.06	–	12
*Prevotella*.7	1	0.08	–	13

The prediction is based on three measures, tabled as mean values, calculated across five inferred co-occurrence NWs (the B, E, T, R and overall network). The mean rank over all possible scoring hierarchies is reported

### *Pseudomonas aeruginosa* and *Staphylococcus aureus* interactions

There is much evidence in the literature that *P. aeruginosa* negatively interacts with *S. aureus*,^39–42^ however, we did not observe a connection between these organisms in the OTU network. Thus, we experimentally investigated the competition between these two species by co-culturing CF isolates together in a CF-lung like environment and comparing that to a *P. aeruginosa* and *Escherichia coli* co-culture as a control (an interaction known to exhibit less antagonism^43, 44^). After equilibration and incubation of a single *P. aeruginosa* strain with three strains of methicillin-sensitive and three strains of methicillin-resistant *S. aureus* together, *P. aeruginosa* was consistently recovered whereas only one *S. aureus* strain survived, further supporting the negative interactions between these two bacteria (Supplementary Fig. S1). In comparison, an incubation of *P. aeruginosa* and *E. coli* resulted in growth and recovery of both strains (Supplementary Fig. S1).

### Function of the CF lung microbiome

To identify the functional structure of the CF community we analyzed an abundance matrix of KEGG modules from twelve previously published and seven new CF metagenomes with co-occurrence networking (‘functional network’). These sputum samples represented all disease conditions, but were from a different patient cohort than the taxonomic analysis. The co-occurrence functional network consisted of seven clusters with more than two nodes (nodes = 50, edges = 79). We then ranked the functional modules (nodes N) according to whether they had the properties of a keystone^37^ based on co-occurrence (Supplementary Table S3). This unsupervised method makes use of topological network properties of the co-occurrence network. In detail, it profiles every node for its node degree (number of interactions), closeness centrality (distance to all other nodes),^45^ and its clustering coefficient (embedment with local neighbors). Then all nodes are ranked by the mean of the three measures shown in Table 2 and the mean rank of all possible hierarchical rankings is used for pinpointing taxa and functions most important to the community. A similar approach to identifying important metabolic functions has been developed by Roume et al.^46^ To compare the two concepts of keystone pathways and key functionalities we adapted the Roume et al.^46^ method to correlation and metagenomic data and compared the results obtained. The ten highest-ranking metabolic modules that were identified as keystones in the functional network are shown in Table 2, and Supplementary Table S3 displays those key functionalities identified as ‘load points’ via the Roume et al.^46^ method. Nine of the 13 keystone KEGG modules identified from the co-occurrence method were also found to have positive load scores according to Roume et al.^46^ (Supplementary Table S3).

**Table 2:** Predicted keystoneness of KEGG module pathways from CF metagenomes

Module	MO	k	Closeness centrality (cc)	Clustering coefficient (clust)	Keystone rank
Putrescine transport	M00300	7	0.052	0.43	1
Glutamate/aspartate transport	M00230	9	0.052	0.31	2
Histidine transport	M00226	8	0.052	0.25	3
Tyrosine biosynthesis	M00025	5	0.052	0.7	4
Pentose phosphate pathway (oxidative phase)	M00006	7	0.053	0.095	5
Ornithine biosynthesis	M00028	9	0.051	0.33	6
Type VI secretion system	M00334	5	0.051	0.5	7
Microcin C transport	M00349	6	0.051	0.33	7
Putative sugar transport	M00197	3	0.052	0.34	7
C5 isoprenoid biosynthesis (non-mevalonate)	M00096	5	0.051	0.8	8
Maltose/maltodextrin transport	M00194	4	0.051	1	8
Dipeptide transport	M00324	4	0.051	0.83	9
Pentose phosphate cycle	M00004	4	0.052	0.17	10

The prediction is based on three measures, the mean rank over all possible scoring hierarchies is reported

**Fig. 2:**
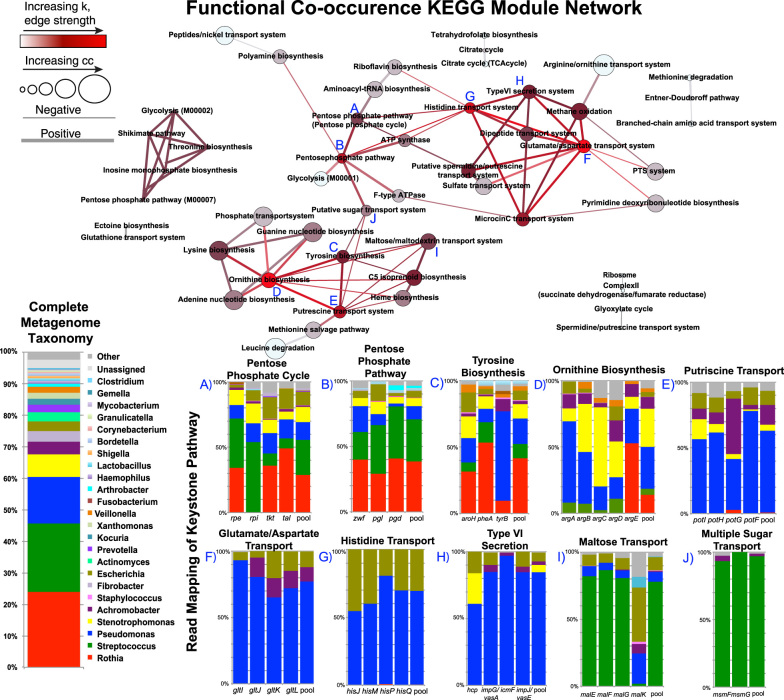
Functional co-occurrence network inferred from the SparCC algorithm applied to KEGG Module abundances from CF sputum metagenomes. Nodes are sized based on their degree of closeness centrality and colored by their number of interactions, where increasing ‘k’ is redder. Edges are sized and colored by the strength of the co-occurrence. Results of the read mapping are highlighted for particular keystone pathways in the network according to the lettering (A–J). The taxonomic distribution of reads to the KEGG module genes is shown as bar graphs of the relative abundance of assigned reads. The relative abundance of taxa from the MG-RAST output of all reads in the pooled metagenomes is shown as a reference

Predicted keystone pathways in the functional network were also subjected to read mapping from genes in these pathways to their bacterium of origin using BLAST (according to the NCBI taxonomy database). This resulted in a phylogenetic overview of taxa with the genetic potential to express keystone metabolic pathways relevant for the entire community structure (Fig. 2). Three of the keystone pathways were involved with the biosynthesis of amino acids, particularly ornithine and tyrosine. Mapping reads to these keystone pathways demonstrated that tyrosine biosynthesis is evenly distributed throughout the community members, but ornithine biosynthesis is mostly associated with *Pseudomonas*, *Stenotrophomonas,* and *Achromobacter* (Fig. 2). Another common group of pathways with the properties of a keystone species were those devoted to the transport of amino acids and putrescine. Reads from the putrescine transport pathway mostly mapped to Climax Community members including *Pseudomonas*, *Stenotrophomonas* and *Achromobacter*. In contrast to the amino acid metabolism keystones, the keystone pathways for maltose/maltodextrin transport and putative sugar transport did not map to Climax Community members (Fig. 2). Reads to this pathway shared sequence homology with *Streptococcus*. It must be noted that the read mapping method utilized in this study cannot account for horizontally transferred genes, where the taxonomic assignment of a read does not match its genetic background.

### Community structure shift during CFPE

**Fig. 3:**
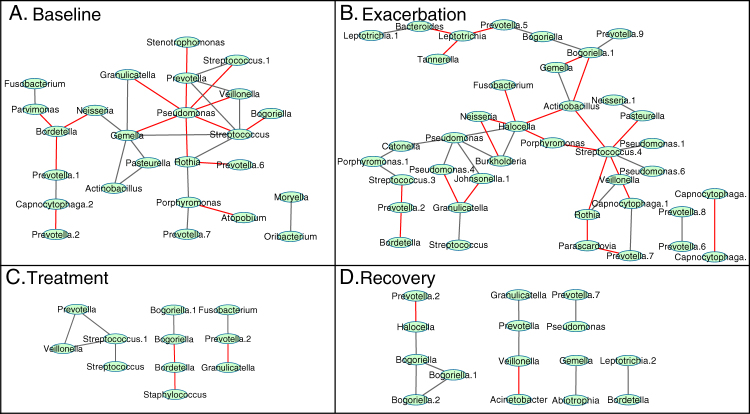
Individual co-occurrence networks of taxa for the BETR categories of disease state. Co-occurrence and anti-occurrence of taxa is denoted with grey lines and red lines, respectively. **a** Baseline network with significant clustering between anaerobes strongly counteracted by *Pseudomonas*. **b** Exacerbation network characterized by re-organization of significant co-occurrence patterns. *Pseudomonas* engages in new specific positive interactions during exacerbation and loses the strong negative effect against the anaerobes seen during stability. **c** Treatment network characterized by small numbers of involved taxa. **d** Recovery network topology breaks into few, sporadic taxon clusters

The CAM predicts that a taxa shift in the microbial community induces a change in the dominant metabolic processes that is associated with CFPE. We tested this hypothesis by applying network analysis to the long-term 16S rRNA gene sputum microbiome data set^38^ separated according to the BETR clinical state categories (‘BETR network’). We obtained four taxa networks, namely one for each clinical state; these are ‘baseline B’, ‘exacerbation E’, ‘treatment T’ and ‘recovery R’ (Fig. 3). To profile the degree of community organization throughout the BETR network, we calculated the network fragmentation *F* that evaluates the formation of topologically independent clusters in the graph. A higher *F* comes from an interaction network with more disconnected clusters relative to the number of nodes.^32^ The co-occurrence patterns changed through different clinical states, resulting in increased *F* of the community during E and R (*F*_B_ = 0.48, *F*_E_ = 0.68, *F*_T_ = 0.53, *F*_R_ = 0.63). T is a singular condition due to increased antibiotic administration and a biased response of the microbial community. The increased community fragmentation during E (and less so in R) is likely caused by an internal restructuring event. Due to the findings of the different core physiologies between the climax and attack communities (Fig. 2) we hypothesize that a microbial metabolism driven drop in pH may be the driver of this restructuring. We subsequently demonstrated that a CF isolate of *P. aeruginosa* favors higher pH, indicating that the climax community is likely the elevated pH group (Supplementary Fig. S2).

**Fig. 4:**
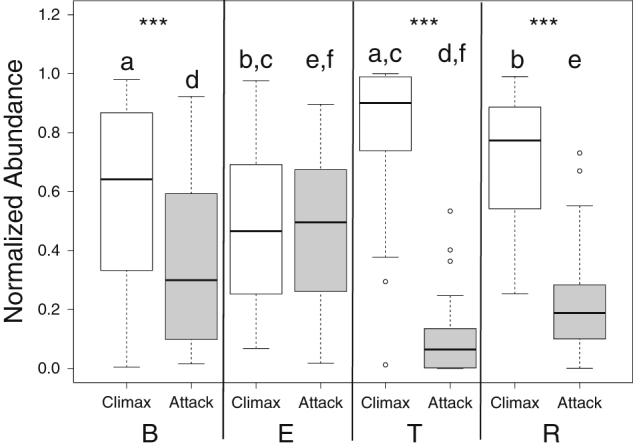
Boxplot distributions of Climax and Attack communities according to assignment of OTU membership from the 16S rRNA gene network topology (see methods). Statistical significance is tested within clinical state using the Student’s *t*-test (Bonferroni corrected *p*-value = *p* < 0.0125, *** = *p* < 0.001) and across clinical states with the Tukey’s test of a one-way ANOVA. Letters designate statistical significances where a shared letter indicates two distributions that were significantly different (a = 0.014, b = 0.04, c = 0.0012, d = 5.7 × 10^−5^, e = 0.0041, f = 4.5 × 10^−6^)

For further support, we evaluated the abundance of Climax and Attack community members across the BETR states in all samples. The original OTU network was used to assign the observed taxa to either of the two communities according to the topological clustering of the graph (Climax communities are the *Pseudomonas* and *Staphylococcus* clusters, *Attack* community is the anaerobic cluster). In Fig. 4, we plotted the normalized abundance of Climax and Attack communities through the B, E, T and R states and tested within clinical state differences with the Student’s *t*-test and across clinical state differences with a Tukey’s test of a one-way ANOVA. The Climax community members were statistically more abundant during all clinical states except for exacerbation, where the attack community increased in its relative abundance to equal the level of the Climax members (Fig. 4, B *p* < 0.001, T *p* < 0.001, R *p* < 0.001, Bonferroni corrected significance *p* = 0.0125). Across disease states the Attack community was significantly higher during E compared to T and R states, but not B (E-T *p* = 4.5 × 10^−6^, E-R *p* = 0.004). The Climax community was significantly more abundant during the T state than B or E (T-B *p* = 0.014, T-E *p* = 0.0011).

### Non-mevalonate pathway of isoprenoid biosynthesis as a novel drug target

**Fig. 5:**
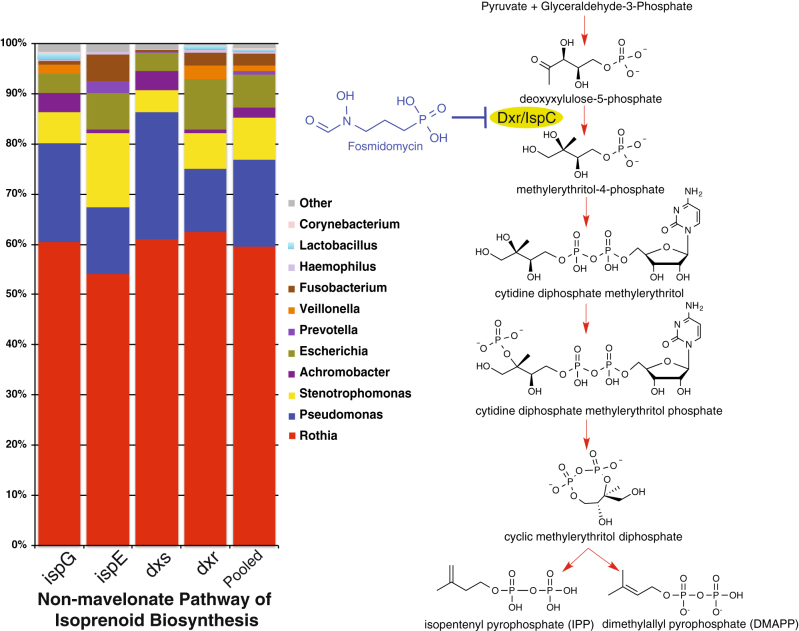
Read mapping of the non-mevalonate pathway reads in CF metagenomes in the context of the chemical species and steps of the pathway. The action of the antibiotic fosmidomycin is shown as a reference

In addition to revealing the important core metabolic pathways of the CF lung microbiome, our functional networking identified that C-5 isoprenoid biosynthesis (non-mevalonate pathway) also had elevated keystone properties (Table 2) from both the co-occurrence method (Table 2) and that of Roume et al.^46^ (Supplementary Table S3). Phylogenetic read mapping of the non-mevalonate pathway genes, including the fosmidomycin target *dxr*, identified a community of bacteria that treatment with fosmidomycin would likely affect directly. The non-mevalonate pathway genes mostly belonged to *Rothia*, but also mapped to *Pseudomonas*, *Stenotrophomonas*, *Achromobacter*, *Escherichia* and the anaerobes *Fusobacterium*, *Veillonella* and *Lactobacillus* (Fig. 5). Thus, we hypothesize that fosmidomycin would be a powerful treatment against *Rothia* spp. and these other bacteria. Although abundant in the metagenomes, none of the reads mapped to *Streptococcus* or *Staphylococcus*, indicating fosmidomycin treatment is not likely to affect these bacteria. This is supported by studies that have shown that these two genera utilize the mammalian mevalonate type of isoprenoid synthesis pathway; an uncommon pathway within the Bacteria.^47, 48^ Furthermore, based on the interactions in the functional network topology (Fig. 2), the use of fosmidomycin in CF could also impact other pathways, as it is positively connected to the maltose/maltodextrin transport and heme biosynthesis. Thus, disrupting isoprenoid synthesis may also inhibit these pathways. *Streptococcus* was the major genus that encoded this sugar transport pathway, indicating that although fosmidomycin may not have a direct effect on these bacteria because they do not encode *dxr* genes, it may indirectly rely on the non-mevalonate pathway or bacteria that use it, making it susceptible, regardless.

To illustrate the percolation of the fosmidmoycin-related effects and predict the reorganization of the CF microbiome after treatment, we implemented a quantitative Lotka-Volterra model.^49^ We first established the microbial metacommunity^50^ that includes relevant taxa from the entire patient cohort, building on the co-occurrence network of the CF taxa (Fig. 1). Fosmidomycin-sensitive taxa (based on the functional read mapping of the non-mevalonate pathway, Fig. 5) were initially removed and the total number of lost organisms recorded. The analysis indicates that the interaction prevalence within the airway microbiome shapes the mean success rate of fosmidomycin treatment (Supplementary Fig. S3A). We hypothesize that as strong interactions increase, potential competitive processes dominate microbiome dynamics, the community composition declines and fosmidomycin percolation decreases. Fosmidomycin percolation (PF) reaches on average 18.3 % (max = 40 %) of the microbial community assessed in a microbiome with 5 % interaction prevalence (Supplementary Fig. S3B). These results suggest that the success of fosmidomycin treatment in targeting potentially unsusceptible organisms of the microbiome crucially depends on the interaction structure of the airway microbiota in the individual patient.

## Discussion

The concept of the keystone originates in community ecology^34^ and identifies a species with disproportionate impact on the community relative to its abundance. Here we applied this keystone concept to microbial abundance data. We also propose the concept of a keystone pathway, upon which the overall community structure and function disproportionately, relies. A similar concept for identifying important enzymes in causal metabolic networks has been proposed by Rahman and Schomberg et al.^51^ So-called ‘choke points’ are characterized by high betweenness centrality (the number of shortest paths that pass through this node) relative to node degree (number of direct interactions) and point to single enzymes that carry out fundamental metabolic conversions in bacteria. Similarly, Roume et al.^46^ analyzed metabolic networks to infer key enzymatic pathways (‘load points’) in microbial communities of biological wastewater treatment plants. To compare the two concepts of keystone pathways^46^ we adapted the Roume et al.^46^ method to our correlation and metagenomic data and compared the results obtained. There was strong congruence between the keystone and ‘load points’ pathways identified, indicating that our methods are in agreement previously established approaches.

**Fig. 6:**
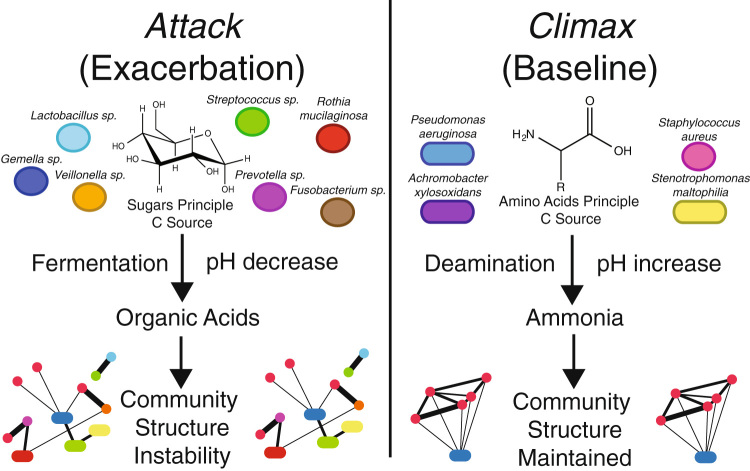
The Climax and Attack Model (CAM) as inferred from co-occurrence network results from this study. Different communities containing fundamentally different physiology affect the surrounding environment differently, principally by changing the pH. This results in community stability during Climax community growth and instability during growth of the Attack community at exacerbation

The progression of CF lung disease is driven by changes in patients’ clinical state, including stable disease (baseline, B), CFPE (E), times of active treatment (T), and recovery from a CFPE (R) (Zhao et al.^38^). The CAM associates these as alternative steady states, similar to those in classical ecology.^21^ The causes of phase shifts between these states is unknown, but hypothesized to be due to changes in microbiome physiological processes and/or antibiotic treatment.^1^ The topology of the OTU network demonstrates that obligate anaerobes are important to the overall community and supports the CAM (Fig. 6). Recent studies of the CF microbiome have identified some anaerobes as important to the pathogenesis of CF lung disease, but they remain a poorly understood group of CF-associated microbes.^24, 52, 53^ Anaerobes and fermentation have been associated with exacerbations of CF.^24, 52, 54^ Fermentative growth by anaerobes lowers the pH of the extracellular environment due to the excretion of acidic fermentation products. This is likely to expand and favor the growth of other fermentative bacteria that are tolerant and thrive in lower pH, such as *Lactobacillus*, *Prevotella*, and *Veillonella*, or facultatively fermentative bacteria, such as *Streptococcus*, *Rothia* and *Granulicatella*.^55–58^ We hypothesize that this pH-based positive feedback loop explains the tight association of anaerobes in the co-occurrence network and may be a driver of shifts between the CAM steady states (Fig. 6). Indeed, the pH of CF lung secretions has been measured as low as 2.9,^59^ is lower than those of healthy controls (exhaled breath condensate average pH: CF = 5.88 and non-CF  = 6.15,^48^), and is lower during exacerbation.^60, 61^ *P. aeruginosa*, however, including strains recovered from CF lungs, does not grow well below pH 5^62^ (Supplementary Fig. S2). Thus, our interpretation of the network topology is that the contrasting physiology of the major bacterial clusters drives the overall structure. The anti-correlation of *Pseudomonas* and the anaerobes (Fig. 1) is an important example. Fermentative anaerobes create microenvironments favorable for their growth to continue, while simultaneously making these environments less suitable for *P. aeruginosa.* A similar pH driven phenomenon has been observed in oral microbial communities,^56^ indicating that core changes in microbial physiology may be a major driver of alternative steady state flux in many polymicrobial systems.

An alternative explanation for the OTU network structure is varying antimicrobial resistance between the two clusters. *P. aeruginosa* is known to be highly resistant to CF antimicrobial therapy,^63^ while CF anaerobes are known to differ greatly in their degree of antibiotic resistance.^52^ The anti-occurrence between *Pseudomonas* and the anaerobes may reflect the presence of antibiotic pressure on the microbial community. *Pseudomonas*’s relative abundance would increase in this situation due to the killing of anaerobic bacteria, which are less resistant to the therapy, explaining the co-occurrence patterns. Another alternative explanation is spatial heterogeneity of microbial distributions in the lung.^64^

*Pseudomonas* is notorious for the production of antimicrobial products, including quinolones, phenazines, and rhamnolipids.^65–70^ These molecules may contribute to negative interactions with the anaerobic group; however, some of these antimicrobials are known to be especially effective against *S. aureus*,^39–42^ with whom a negative correlation was not observed. In laboratory cultures, *P. aeruginosa* readily kills *S. aureus,* ^40–42^ although these species show some synergy in in vitro co-culture models.^71^ The absence of a negative interaction in the OTU network network between these bacteria shows that competitive interactions between *P. aeruginosa* and *S. aureus* are more complex in the lung environment than that of dual species interactions in the laboratory; even when environmental conditions are made similar to the lung. Clearly, microbial interactions are complicated in natural communities, due to the impact of a multitude of other microbes and host factors.^72^

There are biases to consider in the context of our results that are important for any 16S rRNA gene microbiome study of the CF lung. For example, not all of the dominant CF pathogens (e.g., *Burkholderia*) were present in the patients analyzed in this study. The interactions and network position of bacteria not present in these patients is, therefore, unknown. Larger studies incorporating a more comprehensive assessment of the co-occurrences and interactions of all major CF pathogens should be completed to include all significant community members and determine if the structure of the CAM is maintained with a more diverse microbiome. Biases of PCR amplification and DNA extraction that apply to any microbiome study also apply here, potentially confounding some interactions inferred.^38, 73, 74^

The functional network demonstrated that pathways involved in the transport, breakdown and synthesis of amino acids coupled with nitrogenous waste detoxification were keystones for the CF microbial community. The need to synthesize particular amino acids is surprising, as CF sputum is known to be rich in in these compounds,^75, 76^ but this may indicate heterogeneity in the availability of particular amino acids, a trait previously observed.^42^ Transport of amino acids likely reflects their metabolism as a principle carbon source, where histidine transport, dipeptide transport, and glutamate/aspartate transport reflect the import of free amino acids and their subsequent metabolism. Putrescine is also an indicator of amino acid catabolism and nitrogenous waste management. This compound is a common byproduct of amino acid metabolism and is also produced from arginine and ornithine breakdown. Ornithine, a non-essential amino acid, is an important metabolite in the urea cycle. We hypothesize that the keystone importance of synthesizing putrescine and ornithine represents nitrogenous waste management by the lung microbiome. Amino acids are known to be a principle carbon source for CF pathogens^42, 75–78^ and their metabolism has been shown to accumulate waste products such as ammonia.^30^ The results of the read mapping of these pathways supports the idea that amino acid metabolism is a fundamental property of the Climax Community (*Pseudomonas, Achromobacter, Stenotrophomonas* and other classic pathogens). Elevated levels of ammonia have been observed in airway secretions of CF patients and have been shown to contribute to the raising of lung pH, which is normally acidic.^60, 79^ We hypothesize that ammonia production contributes to a stabilization of lung mucus pH for the Climax Community and is a fundamentally important compound for maintaining the stability and community structure of the CF microbial ecosystem.

Other keystone pathways were devoted to the transport and metabolism of sugars and mapped mostly to *Streptococcus* spp. Thus, these bacteria may have a fundamentally different carbon source compared to *Pseudomonas*. Sugar fermentation results in the production of small organic acids that have the opposite effect on the lung environmental pH than ammonia production. Thus, the functional networking in this study indicates that the CF microbial community is partitioned by carbon source. The Climax Community utilizes amino acids, producing ammonia, whereas the Attack Community ferments sugars, producing acids (Fig. 6). Fermentation by *Streptococcus* and anaerobic bacteria has been demonstrated before in CF lung secretions.^24, 54^ This hypothesis is supported by a recent study showing that anaerobic bacteria, first metabolize the sugar component of the mucin polypeptide in CF mucus, before *P. aeruginosa* can subsequently break down its amino acid component.^80^

In light of our results above, we propose that the increased community structure fragmentation observed during exacerbation is due to the differential effects of the core catabolism of the two communities revealed from our functional read mapping. Ammonia production from amino acid breakdown maintains the community at a stable pH, but sugar fermentation by anaerobes, such as *Streptococcus*, disrupts this stable state by lowering the pH of the lung below that favorable for the growth of Climax Community members, such as *Pseudomonas* ^30^ (Fig. 6). Furthermore, analysis of the abundance of Climax and Attack community members in the OTU network revealed that the relative abundance of the Attack community members increased during times of exacerbation. These results strongly support the CAM hypotheses, whereby the Climax community is significantly more abundant in all states except for exacerbation, and during treatment, the Attack community is greatly reduced. In support of this model a recent microbiome study showed that patients with a *P. aeruginosa* as their most dominant taxon had a decreased *P. aeruginosa* abundance at exacerbation.^19^

Finally, an interesting keystone pathway was the non-mevalonate pathway of isoprenoid biosynthesis. This pathway is essential for the synthesis of the basic isoprenoid precursors isopentenyl pyrophosphate and dimethylallyl pyrophosphate.^47, 81^ Such precursors can be synthesized into a highly diverse group of compounds including those important for respiration (ubiquinones) and secondary metabolite production.^81^ Moreover, the respective isoprenoids are produced by unique enzymes indicating the pathway has the inherent characteristics of a ‘choke point’.^51^ Importantly, the enzymes of this pathway are distinct from those of the mammalian mevalonate pathway of isoprenoid synthesis, and have therefore been proposed as novel drug targets.^82^ The identification of this pathway as a significant keystone indicates it is similarly a strong drug target for CF lung infections. Drugs targeting this pathway, such as fosmidomycin,^83^ could directly inhibit the synthesis of isoprenoids and indirectly disrupt the overall community function. Fosmidomycin has mostly been used as an antimalarial,^84^ only rarely being employed as an antibacterial,^85^ but attention is growing around the potential for this drug and others to be developed as a new line of antibacterial treatments.^86^

The finding of the non-mevalonate pathway as a keystone in the CF polymicrobial infection and an antibiotic that targets enzymes in the pathway demonstrates the utility of a functional networking approach to identify novel drug targets against polymicrobial communities. Furthermore, understanding the microbial community interdependencies makes it possible to predict the indirect consequences of a new drug as it further percolates its effect across the microbiota. Finding new ways to treat chronic infections with already approved medicines is very desirable in light of the high prevalence of antibiotic resistant pathogens worldwide.

## Conclusions

More informed antibiotic treatments based on a priori understanding of their effect on a microbial community would result in greater treatment efficacy. To understand the effects of targeting particular pathogens, the underlying structure of a microbial community must be discerned. Here we describe the CF lung microbiome structure and function using ecological co-occurrence networks to better understand the effects of potential antibiotic perturbations. The community is partitioned into an anaerobic group and classic CF pathogens, *Pseudomonas* and *Staphylococcus*. We hypothesize that this structure is driven by the different carbon sources of these groups and the contrasting effect of their metabolism on the pH of the airway environment (Fig. 6). In light of these findings, studies should focus on how simple manipulations of pH may result in the maintenance of a stable CF microbiome and avoidance of the community structure collapse associated with exacerbation. In addition, fosmidomycin should be explored as a novel drug to treat CF infections that may help disrupt overall community interdependencies.

## Methods

Additional methodological information is included in the online Supplementary material.

### Patients and sample collection

Samples for this study had been previously collected for 16S rRNA gene sequencing according to reference 38 and for metagenomics according to Lim et al.^29^ and Whiteson et al*.* ^54^ The clinical state of each patient was classified at the time of sample collection as either at baseline (B), exacerbation (E), during treatment (T) or during recovery from an exacerbation (R) as described (20). All samples were collected in compliance with the University of California Institutional Review Board (HRPP 081500), San Diego State University Institutional Review Board (SDSU IRB#2121), and the University of Michigan Institutional Review Board requirements.

### Data and co-occurrence networks

There were three different co-occurrence network approaches used for this study, a ‘OTU network’ based on 16S rRNA gene data (Fig. 1), a ‘BETR network’ (Fig. 3), which was the same data separated into clinical disease states, and a ‘functional network’ based on metagenomics data (Fig. 2). For the OTU network OTU abundance data generated on 126 sputum samples from 6 different patients in a previously published study^38^ were rarified, OTUs occurring in a single sputum sample only were removed and the resulting 55 OTUs were further used for inferring one correlation network across all BETR categories (Fig. 1). For the ‘BETR network’ the same data was used, but 4 individual co-occurrence networks for each clinical category were built (Fig. 3). For the ‘functional network’ metagenomic data annotated at the level of KEGG metabolic modules using the HUMAnN pipeline^87^ formed the basis of the network (Fig. 2). Data from the 12 454-metagenomes analyzed here were previously published in separate manuscripts with different study objectives and outcomes.^29, 30, 88^ An additional 7 ion torrent metagenomes were included to increase the sample size representing the first publication of this data, except for a targeted analysis of the abundance of a single pathway^54^ that is not described in this study. These data were generated from 19 sputum samples from 10 CF patients, using hypotonic lysis to isolate intact microbial cells before extracting and sequencing the DNA (Supplementary Table S2).^29^ Two different sequencing platforms were used to generate the metagenomic data for networking analysis, the Ion Torrent platform and 454-pyrosequencing (samples with less than 10,000 pyrosequencing reads were not analyzed, sequence read numbers available in Supplementary Table S2). The larger Ion Torrent data were compared against a subset of KEGG gene sequences from several hundred bacterial genomes that have been observed in cystic fibrosis patients, while the 454 data were compared against the entire 2011 KEGG database. The two sequencing and analysis approaches had little influence on the overall data structure as demonstrated by multidimensional scaling of an unsupervised random forest proximity matrix performed in R (Supplementary Fig. S4). Modules present in a single sample only were removed and the resulting 101 KEGG modules (04b file from HUMAnN output) were used to infer the functional network. Due to SparCC requirements, the normalized abundances in the KEGG module output file 04b were adapted by multiplying by the same number (the lowest number of reads in the data set).

For all three networking approaches we inferred co-occurrence networks using the SparCC algorithm^89^ for calculating the correlation strength and significance of all variables against all others (taxa or KEGG modules, respectively). A co-occurrence event was considered for the network, if the correlation > |0.2| and *p*-value ⩽ 0.01. A subsequent correction of FDR according to Benjamini-Hochberg corrected to *p*-values ⩽ 0.003.^90^

### Network fragmentation

To determine the fragmentation pattern of the co-occurrence community, we further simplified the networks and removed anti-correlation edges. Fragmentation was calculated according to F=log(CL)/log*(N)*, where CL is the number of topological clusters in the graph and *N* the number of nodes.^32^ F runs between 0 and 1, where the latter represents an entirely unconnected graph. All analyses were performed in R.

### Keystone prediction

Topological properties of co-occurrence networks can be used to profile keystone constituents according to reference 37. These properties include the node degree *k*, closeness centrality *cc* and the clustering coefficient *clust*. We used the species BETR network to filter for a list of correlating species. Then, we evaluated each species for the three topological properties in every individual network (B, E, T, R) and calculated mean k (<k>), mean cc (<cc>) and mean *clust* (<clust>). To profile keystone species we ranked each species taking into account all six permutations of the three properties. Networks excluding and including anti-correlations were analyzed (Table 1, Supplementary Table S1, respectively). The metabolic modules were similarly tested for their importance within the community by querying the BETR network for the analysis.

### Load point prediction

Load scores of all nodes in the metabolic co-occurrence network were calculated according to Roume et al.:^46^

$${\rm{load}}\,{\rm{scor}}{{\mathrm{e}}_n} = \frac{\mathop{\sum}\nolimits_{s \ne n \ne t}\left.\left(\sigma _{st}(n)\right)/\sigma_{st}\right)}{\frac{k_n}{\sum e }},$$

where the score of node n is calculated from all shortest paths *σ* between any node *s* and *t* in the graph that pass through node *n*, and is normalized to the relative degree of node n. The relative degree is the ratio of the number of edges of node n versus the total edge number e in the graph.

While in the original study connectivity-centered metabolic networks that group enzymes by similar KOs as nodes and use metabolites as undirected edges were analyzed, we performed the identification of load points on co-occurrence networks of KEGG modules. In Roume et al.^46^ further prioritizing of the nodes is performed calculating relative expression of genes from metatranscriptome data. Here, we summarize the normalized read abundance of KEGG modules in metagenomic samples as semi-quantitative information along with load score in Supplementary Table S3.

### Abundance shift

Sub-groups of species were formed according to topological properties of the species OTU network. The network was split along negative correlation into 2 groups designated Climax and Attack. The A group included the *Attack* community members *Prevotella*, all *Streptococcus* OTUs, *Veillonella*, *Fusobacterium*, *Gemella*, *Granulicatella,* and all other members of the anaerobic cluster while the Climax group included *Bogoriella*.1, *Halocella*, *Bogoriella*, *Staphylococcus*, and *Pseudomonas*. Relative abundances were collected and analyzed for statistical differences within clinical state (Student’s *t*-test, Bonferroni corrected) and across clinical states (Tukey’s test of one-way ANOVA). All analyses were performed in R.

### Functional read mapping

KEGG Modules predicted to have strong importance for the functional network were selected for read mapping to the most closely related organism of individual genes that made up the module. This read mapping was done for 10 total pathways and compared to the taxonomic distribution of the entire pooled metagenome, to get an impression of the taxonomic distribution of particular pathways relative to the taxonomy of the entire community. The read mapping was done on individual genes in each pathway and then pooled for an overall pathway comparison. The 454-pyrosequencing and Ion Torrent metagenomes were pooled into two separate fasta files and uploaded to the MG-RAST server separately, in order to allow downloading of individual reads to genes of interest. The complete taxonomic distribution of the entire data set was determined using the lowest common ancestor feature of the MG-RAST pipeline.^91^ Each individual read mapping to a particular gene that made up a keystone KEGG module of interest was retrieved using the MG-RAST workbook feature. These reads were then collectively searched against the GenBank database using BLAST. The top BLAST hit for each read was extracted and its organism of origin determined from the NCBI taxonomy database.

### Fosmidomycin percolation

To assess how the application of fosmidomycin percolates throughout the microbial community we implemented a generalized Lotka-Volterra ODE model with the aim of quantifying the loss of taxa after treatment. The CF metacommunity of microbial taxa^85^ was established from the co-occurrence relationship inferred from the patient cohort. We repeatedly subsampled on average 20 taxa and their interactions from the CF metacommunity for obtaining individual patient microbiomes and simulated the time evolution of the organisms until they reached equilibrium. We assessed the percolation of fosmidomycin in communities with 5 % interaction prevalence (Supplementary Fig. S3B). For assessing the impact of interaction organization on fosmidomycin percolation (Supplementary Fig. S3A), we conducted simulations for 900 microbiomes with varying interaction prevalence (0–45 %). For each prevalence bin, we generated 10 realizations of the metacommunity and therefrom subsampled and simulated 10 individual microbiomes. The number of drug related direct and indirect kills was recorded and normalized against community size without treatment. Further details of the percolation method are described in the online Supplementary methods.

### Bacterial culture experiments

Strains used for this study were isolated from sputum samples at the University of California at San Diego Center for Advanced Laboratory Medicine. The co-culture experiments involved three methicillin sensitive *S. aureus* strains (SaFLR01, SaFLR02, and SaFLR03), three methicillin resistant *S. aureus* strains (MRSAFLR01, MRSAFLR02, and MRSAFLR03), and an *E. coli* isolate (EcFLR01) all grown separately in co-culture with a *P. aeruginosa* CF isolate (PAnmFLR01). After CFU equilibration, 25 μl of each culture was added to 500 μl of artificial sputum medium (ASMRQ formulation) and inoculated into non-heparinized glass capillary tubes for growth (Fisher Scientific) (for method see Quinn et al.^24^). After 24 h incubation at 37 °C the media was serially diluted in phosphate buffered saline and unique colonies were counted on Todd Hewitt agar. The *P. aeruginosa, S. aureus* and *E. coli* bacteria produced morphologically unique colonies allowing for a determination of individual counts of each species.

## Electronic supplementary material

Supplementary Information

Supplementary Tables

Supplementary Figure 1

Supplementary Figure 2

Supplementary Figure 3

Supplementary Figure 4
