# Supplementary material for: Ecological networking of cystic fibrosis lung infections
Source: NPJ Biofilms Microbiomes. 2016 Dec 2;2:4. doi: 10.1038/s41522-016-0002-1 (PMC5460249; doi:10.1038/s41522-016-0002-1)
Supplement: Supplementary file 1 — Supplementary Information [file 41522_2016_2_MOESM1_ESM.docx]

**Supplementary Methods:**

*Patients and sample collection:* Sputum samples for 16S rRNA gene sequencing were collected during routine clinical care at the University of Michigan Health System clinical microbiology laboratory and stored at -80°C. Sputum samples for metagenomics were collected at the University of California Adult Cystic Fibrosis clinic using inhaled 7% hypertonic saline after an oral saline rinse. Samples were collected for 30 minutes during saline inhalation, homogenized with a syringe, and kept on ice until processing within 24 hours.

*Functional read mapping to determine taxonomic distributions:* The 454-pyrosequencing pooled metagenome contained 654,107 reads (average read length was 413 +/- 103 bp) and the Ion Torrent pooled metagenome contained 14,504,987 reads (average read length average read length of 137 +/- 40 bp). Both datasets were uploaded to MG-RAST, and the number of reads to any gene from the Ion Torrent file was normalized to proportional difference in total read abundance of the smaller 454-pyrosequencing data file to allow comparison of the number of hits across sequence files. The taxonomic distribution of all the reads for a specific KO was counted at the level of genus and then summed across both files after read normalization and visualized as a bar graph.

*Bacterial culture experiments:* All bacteria were grown to 1.0 x 10^6^ cfu/ml in Todd Hewitt Broth to equilibrate their cell density for competition. The WinCF culture model was used for these competition experiments to better mimic the environment of the CF lung (Quinn *et al.*, 2015). To determine the pH tolerance of *P. aeruginosa* CF isolate PAnmFLR01 was grown in LB broth at pH 5, 6, 7, 8 and 9 in a 37°C shaker incubator at 200 rpm. Aliquots of 1ml of the media were taken at different time intervals up to 68 hours and optical density measurements were read in a spectrophotometer at 600 nm.

*Fosmidomycin percolation:* To assess how the application of fosmidomycin percolates throughout the microbial community we implemented a generalized Lotka-Volterra ODE model with the aim of quantifying the loss of taxa after treatment. The principal assumption is that the removal of taxa that are sensitive to fosmidomycin will affect other taxa that are linked by interactions and are otherwise potentially insensitive to the drug.

*ODE model*: The Lotka-Volterra model^84^ that is growth limited by a global carrying capacity K_g_ writes as follows:

The abundance x of taxon i is calculated as function of its growth rate r_i_, the community-wide interactions A_i_ with taxon i, the individual carrying capacity k_i_ and the global carrying capacity K_g_.

*CF metacommunity*: The CF metacommunity of microbial taxa^85^ was established from the co-occurrence relationship inferred from the patient cohort. Correlation was conducted using SparCC as described in Materials and Methods, p-value cutoff was set to p<0.1 after false discovery correction according to Benjamini-Hochberg. Recovered correlation strengths where unified to 1 and -1 for positive and negative correlations, respectively, only keeping the overall organization for further experiments. The CF metacommunity consists of 55 taxa and 426 potential interactions (positive: negative correlations = 214:212).

*Simulations*: We repeatedly subsampled on average 20 taxa and their interactions from the CF metacommunity for obtaining individual patient microbiomes and simulated the time evolution of the organisms until they reached equilibrium. Growth rates r, carrying capacities k and initial conditions were sampled from a random distribution, interactions were either present or absent (1|0), Kg was set to 10000. All simulations were performed in R. We assessed the collective effect of fosmidomycin percolation in the communities by counting killed taxa (abundance < 0.0001) after the removal of drug sensitive taxa.

*Percolation analysis*: We assessed the percolation of fosmidomycin in communities with 5% interaction prevalence (Figure S4B). To that aim we generated 10 independent realizations of 5% metacommunities by randomly selecting 5% interactions of the original CF metacommunity and setting all other interactions to 0. Each of these realizations were subsampled 10 times to generate individual patient microbiomes subjected to simulation. The number of killed taxa upon treatment (=removal of susceptible taxa) was collected and normalized against community size without treatment. The probability p(PF) for a percolation to spread across a certain percentage x of the microbial community was calculated as (N - Σ_cum_ N_x_)/N, where N is the number of all cases in the experiment and Σ_cum_ N_x_ is the cumulative sum of cases where 0-x % of the community were killed.

For assessing the impact of interaction organization on fosmidomycin percolation (Figure S4A), we conducted simulations for 900 microbiomes with varying interaction prevalence (0-45%). For each prevalence bin, we generated 10 realizations of the metacommunity and therefrom subsampled and simulated 10 individual microbiomes. The number of drug related direct and indirect kills was recorded and normalized against community size without treatment.
