# Supplementary material for: Ecological networking of cystic fibrosis lung infections
Source: NPJ Biofilms Microbiomes. 2016 Dec 2;2:4. doi: 10.1038/s41522-016-0002-1 (PMC5460249; doi:10.1038/s41522-016-0002-1)
Supplement: Supplementary file 2 — Supplementary Tables [file 41522_2016_2_MOESM2_ESM.docx]

Table S1. Keystone prediction of 16S rDNA community after inclusion of both negative and positive interactions.

| OTU | k | Closeness Centrality (*cc*) | Clustering Coefficient (*clust*) | Keystone Rank |
| --- | --- | --- | --- | --- |
| *Streptococcus*.1 | 4.43 | 0.3 | 0.623 | 1 |
| *Gemella* | 4.75 | 0.26 | 0.53 | 1 |
| *Streptococcus* | 4.5 | 0.27 | 0.43 | 2 |
| *Veillonella* | 3.2 | 0.2 | 0.64 | 3 |
| *Fusobacterium* | 2.25 | 0.23 | 0.67 | 4 |
| *Prevotella* | 4 | 0.22 | 0.49 | 5 |
| *Pseudomonas* | 4.5 | 0.22 | 0.34 | 6 |
| *Granulicatella* | 3 | 0.20 | 0.5 | 7 |
| *Bogoriella*.1 | 2.25 | 0.17 | 0.72 | 8 |
| *Bogoriella* | 2.4 | 0.19 | 0.17 | 9 |
| *Halocella* | 3 | 0.18 | 0.07 | 10 |
| *Bordetella* | 1.75 | 0.12 | 0 | 11 |
| *Prevoltella*.2 | 1.34 | 0.11 | 0 | 12 |
| *Prevotella*.7 | 1.34 | 0.11 | 0 | 12 |

Table S2. Disease state and sequencing information of metagenomes analyzed in this study.

| Patient ID | Disease State | Sequence reads | Sequencing Platform |
| --- | --- | --- | --- |
| CF1 | B | 1,494,421 | Ion Torrent |
| CF1 | E | 22147 | 454 Pyrosequencing |
| CF1 | T | 78200 | 454 Pyrosequencing |
| CF1 | R | 43417 | 454 Pyrosequencing |
| CF10 | R | 1,428,012 | Ion Torrent |
| CF11 | B | 1,436,594 | Ion Torrent |
| CF12 | B | 964,098 | Ion Torrent |
| CF13 | E | 1,471,073 | Ion Torrent |
| CF4 | B | 59629 | 454 Pyrosequencing |
| CF6 | B | 1,973,912 | Ion Torrent |
| CF6 | T | 80745 | 454 Pyrosequencing |
| CF6 | R | 41146 | 454 Pyrosequencing |
| CF7 | E | 49446 | 454 Pyrosequencing |
| CF7 | T | 33260 | 454 Pyrosequencing |
| CF7 | T | 14187 | 454 Pyrosequencing |
| CF7 | R | 32728 | 454 Pyrosequencing |
| CF8 | E | 142564 | 454 Pyrosequencing |
| CF8 | R | 34429 | 454 Pyrosequencing |
| CF9 | B | 4,334,671 | Ion Torrent |

Table S3. Pathways encoding key functionalities according to the method of Roume et al. (2015). The same data used for keystone prediction based on co-occurrence was used for ‘load point’ calculation. Relative abundance of each module is also shown. Keystone pathways also identified using the co-occurrence method are highlighted in bold.

| Module ID | Module Name | Relative Abundance | Load |
| --- | --- | --- | --- |
| **M00004** | Pentose phosphate pathway (Pentose phosphate cycle) | 0.020 | 195 |
| **M00300** | Putrescine transport system | 0.006 | 156 |
| **M00006** | Pentose phosphate pathway | 0.023 | 140.4 |
| M00034 | Methionine salvage pathway | 0.005 | 136.5 |
| M00360 | Aminoacyl-tRNA biosynthesis | 0.027 | 117 |
| **M00028** | Ornithine biosynthesis | 0.008 | 110.5 |
| M00174 | Methane oxidation | 0.003 | 70.688 |
| **M00230** | Glutamate/aspartate transport system | 0.002 | 56.179 |
| **M00334** | TypeVI secretion system | 0.001 | 26 |
| **M00096** | C5 isoprenoid biosynthesis | 0.012 | 19.5 |
| **M00349** | MicrocinC transport system | 0.002 | 13 |
| M00016 | Lysine biosynthesis | 0.015 | 9.75 |
| **M00193** | Putative spermidine/putrescine transport system | 0.004 | 6.5 |
| **M00226** | Histidine transport system | 0.007 | 2.438 |
| M00324 | Dipeptide transport system | 0.001 | 2.438 |
| M00001 | Glycolysis (Embden-Meyerhof pathway) | 0.029 | 1.00E-04 |
| M00049 | Adenine nucleotide biosynthesis | 0.008 | 1.00E-04 |
| M00050 | Guanine nucleotide biosynthesis | 0.003 | 1.00E-04 |
| M00222 | Phosphate transport system | 0.022 | 1.00E-04 |
| M00125 | Riboflavin biosynthesis | 0.021 | 1.00E-04 |
| M00025 | Tyrosine biosynthesis | 0.016 | 1.00E-04 |
| M00194 | Maltose/maltodextrin transport system | 0.015 | 1.00E-04 |
| M00197 | Putative sugar transport system | 0.024 | 1.00E-04 |
| M00121 | Heme biosynthesis | 0.024 | 1.00E-04 |
| M00036 | Leucine degradation | 0.003 | 1.00E-04 |
| M00157 | F-type ATPase | 0.008 | 1.00E-04 |
| M00164 | ATP synthase | 0.009 | 1.00E-04 |
| M00235 | Arginine/ornithine transport system | 0.022 | 1.00E-04 |
| M00185 | Sulfate transport system | 0.000 | 1.00E-04 |
