# Supplementary figures and images for: Ecological networking of cystic fibrosis lung infections
Source: NPJ Biofilms Microbiomes. 2016 Dec 2;2:4. doi: 10.1038/s41522-016-0002-1 (PMC5460249; doi:10.1038/s41522-016-0002-1)

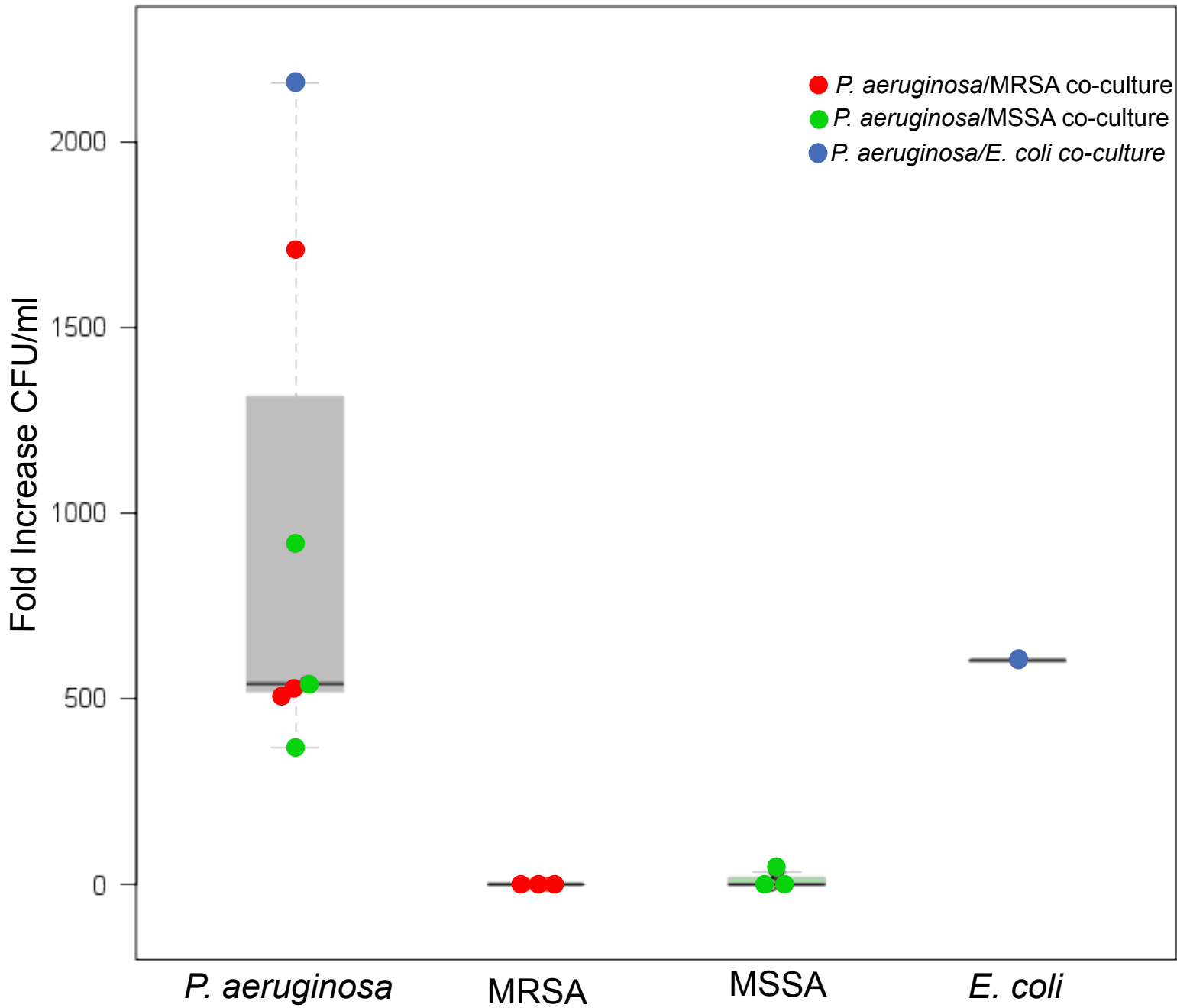

Supplement: Supplementary file 3 — Supplementary Figure 1 [file 41522_2016_2_MOESM3_ESM.pdf]

Optical Density

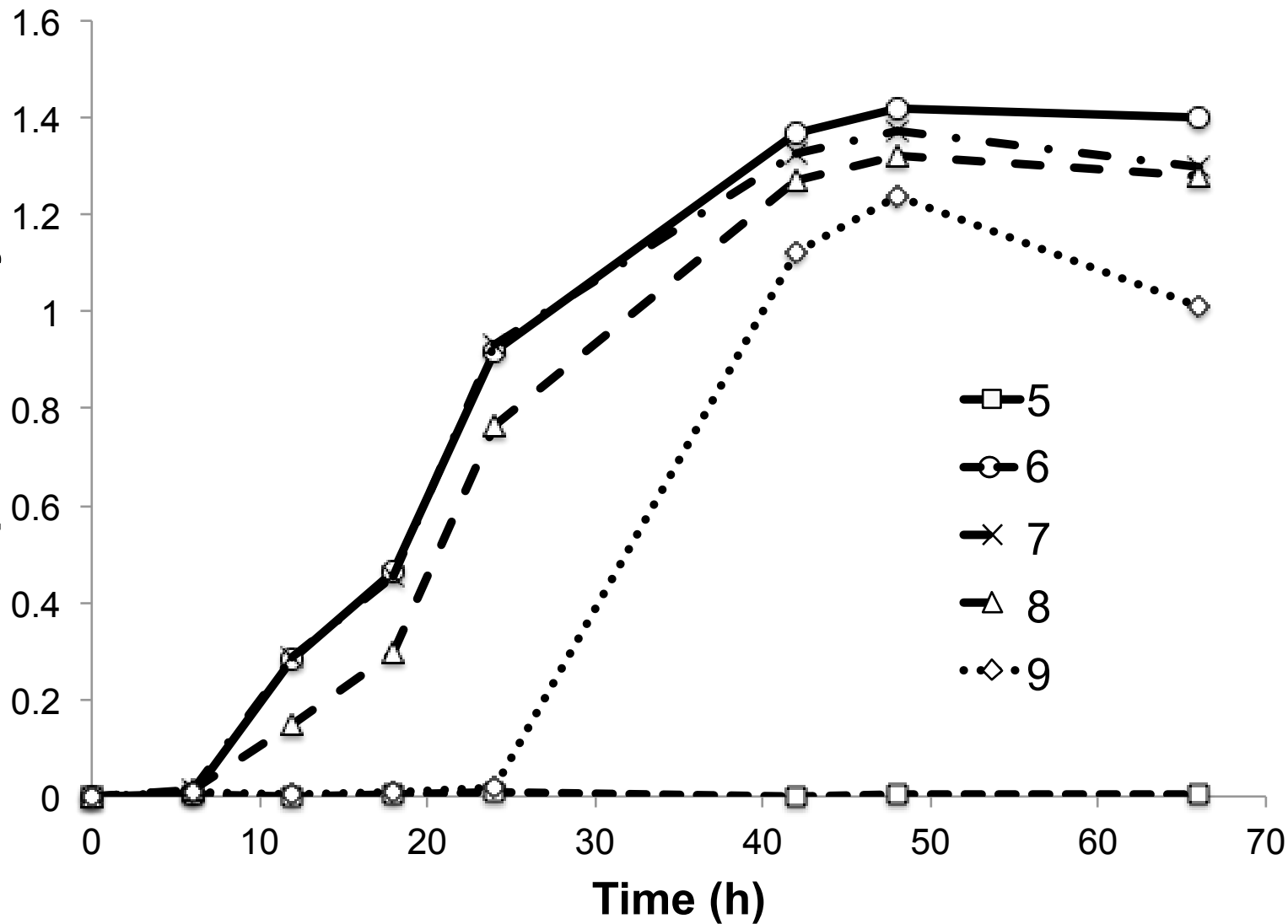

Supplement: Supplementary file 4 — Supplementary Figure 2 [file 41522_2016_2_MOESM4_ESM.pdf]

**A**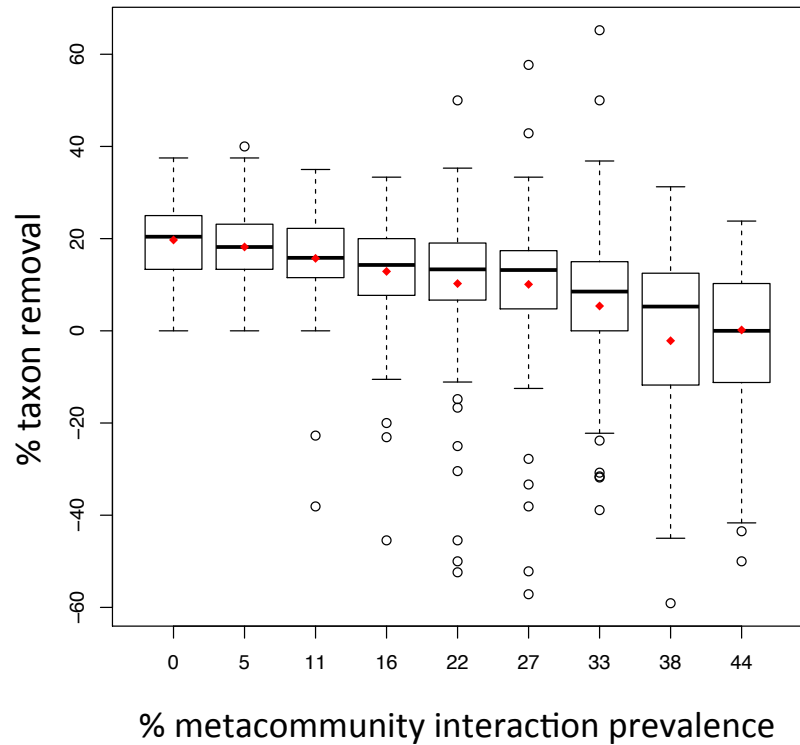**B**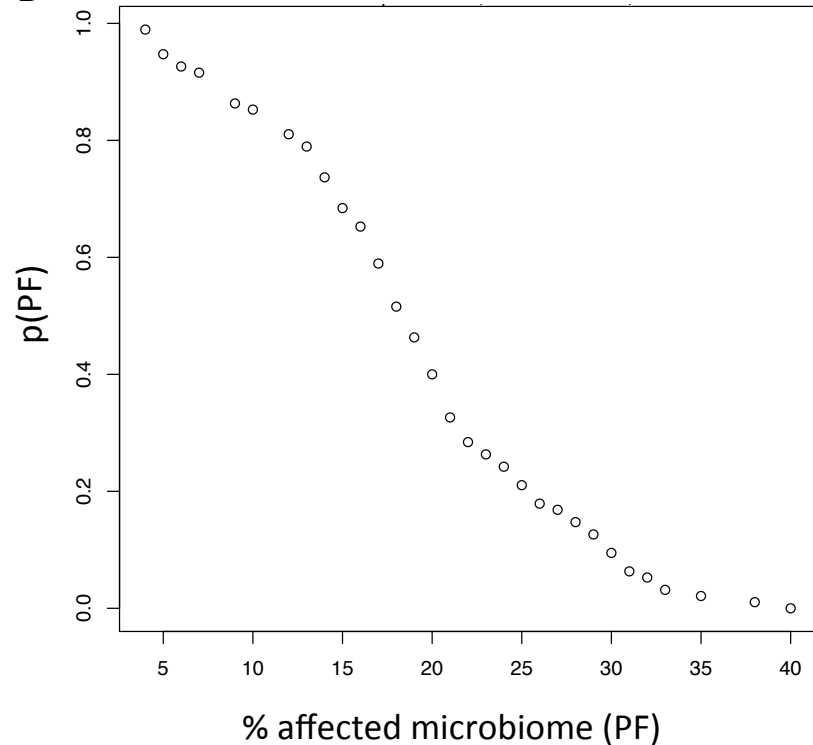

Supplement: Supplementary file 5 — Supplementary Figure 3 [file 41522_2016_2_MOESM5_ESM.pdf]

# CF Metagenome Random Forest

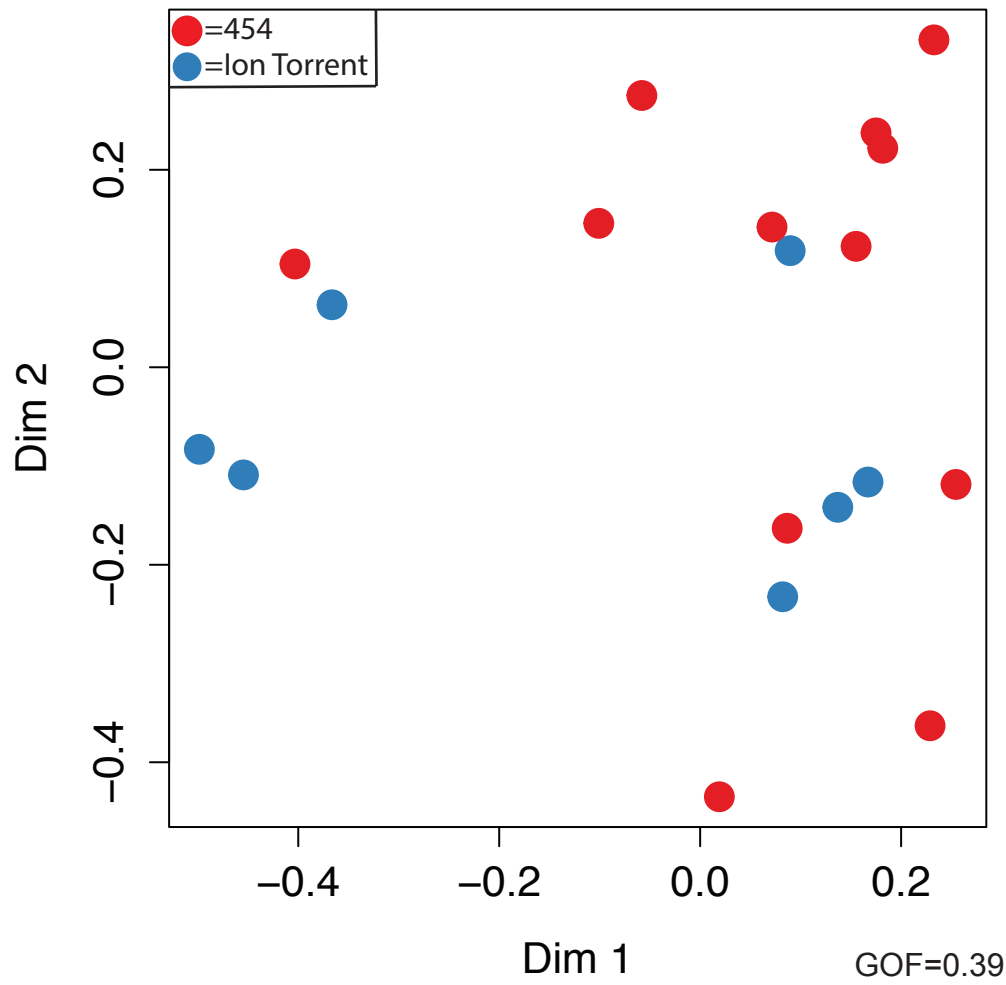

Supplement: Supplementary file 6 — Supplementary Figure 4 [file 41522_2016_2_MOESM6_ESM.pdf]
